# Food safety behaviour and handling practices among Saudi women during the COVID-19 pandemic

**DOI:** 10.6026/97320630017870

**Published:** 2021-10-31

**Authors:** Leila Arfaoui, Wejdan Alghafari

**Affiliations:** 1 Clinical Nutrition Department, Faculty of Applied Medical Sciences, King Abdulaziz University, Jeddah, Saudi Arabia

**Keywords:** Purchasing habits, food safety practices, food safety behaviour, COVID-19, Saudi women

## Abstract

Although it is commonly known that viruses cannot multiply in food as they need a living host for growth, adenoviruses and corona viruses can reportedly survive on surfaces and food packages for several days. Therefore, food item mishandling could increase the risk of infection. In this cross-sectional study, we assessed the changes in the food purchasing habits, food safety behavior, and food handling practices among Saudi women during the COVID-19 pandemic. The study included 1356 women who were randomly approached via convenience sampling using an anonymous questionnaire distributed through various social media platforms. The variables were described in terms of frequency and percentage, and the Chi-square test was applied to assess the relationship between the dependent and independent variables. Approximately 62.5% of the participants were aware that SARS-CoV-2 is not transmitted via food. Most participants (90%) reported a shift from outdoor to indoor meal preparation, along with avoidance of visits to grocery stores for food purchase (55%) and increased usage of online grocery delivery services (27%). Most participants obtained good overall scores for food safety behavior during grocery shopping (mean score: 10.83±1.62/12 points, 90.25%), grocery unpacking at home (10.60±2.65/13 points, 81.55%), and personal hygiene (28.84±3.16/36 points, 80%). However, a moderate overall score was obtained for food preparation practices (7.77 ±1.91/12 points, 77.7%). Older and/or retired individuals, patients with chronic diseases, and/or individuals living with children showed better food safety behavior and handling practices compared to their counterparts. This study reported good overall food safety behavior and handling practice scores among participants under most categories studied. However, our results highlight the need for more customized public education programs for Saudi women, who are the primary food handlers in most households, particularly during food preparation, to further improve food safety practices and prevent potential food mishandling, which will eventually help preventing the spread of COVID-19.

## Background:

The severe acute respiratory syndrome corona virus 2 (SARS-CoV-2) is the causative agent in corona virus disease (COVID-19), an infectious respiratory disease in humans [1,2]. Corona viruses are highly transmissible from patients to healthy individuals via respiratory droplets released during coughing, sneezing, or exhalation and via surface contact routes. To date, there have been no evidences of SARS-CoV-2 transmission through food or food packaging [3]. However, the Center for Disease Control and Prevention of China isolated a live SARS-CoV-2 viral genome from the packaging material of seafood and chicken wings imported from Brazil [4]. The virus remains viable for up to 72 h depending on the surface type, humidity, and temperature of the environment. For example, SARS-CoV-2 survived for 3 and 2 days on plastic and stainless-steel surfaces, respectively, at 21–23°C and 40% relative humidity, but it was not detectable on cardboard and copper surfaces after 24 and 4 h, respectively [5]. As corona viruses can survive on different surfaces for more than a few days, they can be potentially transmitted to the human body via contact with contaminated surfaces (including food packages/containers), as proposed by the WHO and Food and Agriculture Organization. Several respiratory viruses (including SARS-CoV-1 and 2 and influenza A and B/H1N1) can spread via plausible transmission sources in the environment, such as food products [6]. Imported palm oil, cashews, and cocoa beans from South Africa were considered potential contaminants and transmitters of Ebola virus to the human population in the United States; however, this effect was estimated to be negligible [7]. Cross-contaminated food and foods originating from infected animals have been proposed as potential sources of food borne SARS-CoV-2 transmission to humans [8]. Although it is broadly recognized that viruses cannot multiply in food as they require a living host (human or animal) for growth, adenoviruses and corona viruses have been reported to survive on the surface of some food items, such as lettuce and strawberries, for several days [9]. Even at a considerably low titer, most respiratory viruses can be infectious; therefore, the methods used for the handling and consumption of food items could increase the risk of infection. As SARS-CoV-2 can survive for 72 h as a virion on non-living objects after completing its life cycle in the living human host, it can function as a fomite and can infect other individuals who touch the food material/packaging contaminated with the respiratory discharges of patients with COVID-19. The virus enters the respiratory epithelium via contact between the hands and the mouth, nose, or eyes [10]. Therefore, preventive measures, such as appropriate personal hygiene habits and behavior during shopping and grocery unpacking, as well as adherence to proper guidelines related to food preparation and handling, could be effective methods to prevent COVID-19. Domestic kitchens are considered a critical point for the spread of food-borne diseases owing to the lack of food safety practices among consumers, especially during the ongoing pandemic. In Saudi Arabia, women are considered as the chief food handlers since they are mostly engaged in cooking for their family. Therefore, their behavioral changes in response to the COVID-19 pandemic and their food handling practices should be assessed. It is of interest to identify non-compliance to safe food handling practices and behavior under the categories of food shopping, food unpacking, food handling and preparation, and personal hygiene to design programs for educating Saudi women on safeguarding practices for themselves and their families during and after the COVID-19 crisis.

## Methods:

### Study design and participants:

In this cross-sectional study, 1356 women above 18 years of age agreed to participate. Women were randomly approached using convenience sampling with an anonymous online questionnaire distributed through various social media platforms from July 15 to September 15, 2020. The questionnaire first briefed the participants on the purpose of the study and the estimated time for questionnaire completion and asked the participants a yes/no question on their consent to participate in the study. Only participants who provided consent received access to the complete questionnaire. Ethical approval was obtained from the Research Ethics Committee of the KAU Hospital (Ref. number: 496-20).

### Sample size calculation:

The sample size was calculated using the online software Epi Info sample size calculator for epidemiologic statistics supported by the Division of Health Informatics & Surveillance, and Center for Surveillance, Epidemiology and Laboratory Services (https://www.openepi.com/SampleSize/SSPropor.htm) [11]. The data were obtained from the Saudi General Authority for Statistics (2019) and included information on an estimated 10,362,080 women (aged >18 years). The effective sample size for this study was 1083 participants, with a 99.9% confidence interval, an effect size of 1, α error probability of 0.05, and a 50% hypothesized frequency of outcome factor in the population.

### Questionnaire and data collection:

The questionnaire was designed by three food science experts and consisted of seven sections. Thirty-eight questions were included from three different sources.

(i) The first section of the questionnaire comprised ten questions assessing the characteristics of the participants, including socio demographic characteristics (age group, marital status, educational level, occupation, income level, and presence/absence of children), health status (presence of any chronic disease), and presence of elderly individuals or individuals with chronic disease(s) at home.

(ii) The second section comprised three questions on whether the COVID-19 virus could be transmitted by food, whether proper handwashing routine of 40 s with soap and water can reduce the risk of corona virus transmission, and the source of information used for learning about preventive measures to limit the spread of new corona virus strains.

(iii) The third section of the questionnaire comprised seven questions that assessed the effect of the COVID-19 crisis on food purchasing habits; a questionnaire prepared by the International Food Information Council was used here [12].

The fourth (iv), fifth (v), and sixth (vi) sections assessed the behaviour of the participants during grocery shopping, grocery unpacking, and personal hygiene during the COVID-19 pandemic, respectively, through 14 frequency-based questions defined on the basis of the precautions reported by Desai and Aronoff on food safety and COVID-19 [13]. Participants had to choose from four responses (never, rarely, sometimes, and usually), and the responses were scored from 0 to 3, with higher scores indicating better safety behaviour/personal hygiene. The seventh section (vii) of the questionnaire included four questions on food preparation practices in domestic kitchens during COVID-19, which were adopted from a study by Ayaz et al. [14]. These questions had four responses, and each response was provided a score from 0 to 3, with higher scores indicating safer practices. The questionnaire was subjected to face validation and pretesting and improvised accordingly. An online survey form was created using Google Forms and distributed via various social media applications (such as WhatsApp and Twitter) and via email.

### Statistical analysis:

Food purchasing habits, grocery shopping behaviour, practices followed during grocery unpacking at home, food preparation practices, and personal hygiene during the COVID-19 pandemic were the dependent variables. These variables were scored in a manner such that a higher score corresponded to safer behaviour/personal hygiene/practices. The total score of each section and its median value were computed and categorized as "low" (<50th percentile), "intermediate" (50th–75thpercentile), and "good" (>75thpercentile). The independent variables included age, marital status, presence/absence of children, educational level, domain of employment, monthly income, diagnosis of chronic diseases, and presence of elderly or chronically ill individuals at home. Frequency and percentage were used to describe the variables, and the Chi-square test was applied for inferential statistics to assess the relationship between the dependent and independent variables, with p<0.05 considered statistically significant. Data entry and analyses were performed using the Statistical Package for Social Sciences version 25(SPSS Inc., Chicago, IL, USA).

## Results:

### Socio demographic characteristics of participants and their knowledge on corona virus transmission:

Of the 1369 women enrolled in our study, 1356 responded to our survey, yielding a response rate of 99.05%. The demographic and personal characteristics of the participants are outlined in Table 1(see PDF). Almost one-third of the women were aged between 26 and 35 years, whereas only 5.8% of them were older than 55 years. Most participants were married (69.5%) and had children (69.3%). Almost two-thirds of the participants were university graduates (65.7%), and more than one-third of the participants were unemployed (37.8%), whereas 15.2% were employees in the health domain and 24.9% in other domains. Almost one-third of the participants (33.8%) had no income, whereas 23.2% had an income ranging between 5000 and 10000 SR/month. In the study population, 31% individuals were diagnosed with chronic diseases, 40.6% reported having elderly individuals at home, and 52% had patients with chronic diseases at home.

Almost two-third of the study population (62.5%) responded that corona virus is not transmissible via food, while the majority (97.6%) recognized that proper hand washing with soap and water for 40 s is important for reducing the risk of corona virus transmission. Reportedly, the primary source of information used to learn about preventive measures to limit the spread of SARS-CoV-2 infections was the website of the Saudi Ministry of Health (MOH) (75%), followed by social media (45.5%), as shown in Figure 1(see PDF).

### Food purchasing habits:

The changes in food purchasing habits due to the COVID-19 pandemic among the participants are presented in Table 2(see PDF). The majority of women ordered food and beverage less frequently or did not order the same during the pandemic (90.4% and 91.4%, respectively). Accordingly, almost the same proportion of participants reported an increased frequency of meal preparation at home (90.4%). Almost one-fifth of the participants reported an increase in the purchase of canned, frozen, and pre-packed food items, whereas the majority of them either purchased these products at a similar frequency as earlier or at a lower frequency. Almost 60% of the participants reported that they bought more fresh fruits and vegetables during the COVID-19 pandemic. Half of the participants reported visiting grocery shops less frequently. Although approximately 30% of the participants reported using online grocery ordering services more frequently than before, almost half (43.3%) of them did not use this service at all.

### Behaviour during grocery shopping:

Most participants reported wearing a mask during shopping (92.3%), maintaining a distance of at least 2meters with other shoppers (80.2%), frequently sanitizing their hands during shopping (77.9%), and wiping the basket/trolley with a sanitizer before using it for shopping (65.6%) as shown in Table 3(see PDF). The average score in this section was 10.83±1.62/12 points (90.25%), indicating that most participants adhered to the recommended measures during grocery shopping.

### Behaviour during grocery unpacking at home:

Table 3(see PDF) shows that most participants reported unpacking grocery bags on a separate countertop away from other kitchen items (64.6%), cleaning and sanitizing the countertop and all other surfaces used for unpacking (64.8%), cleaning canned food containers (74.8%), and immediately storing perishable/frozen food (84.7%) in the refrigerator or freezer. However, only 13.2% participants reported storing raw meat on the bottom shelf of the refrigerator. The average score for this section was 10.60%±2.65% with a maximum score of 13 points (81.55%), which indicates overall good behaviour among participants during grocery unpacking at home.

### Personal hygiene practices

With respect to hand hygiene, most participants reported washing their hands after visiting public places (94.5%), after unpacking grocery bags (89.8%), before eating food (89%), and before and after handling food (85.7%). A lower proportion of participants reported washing their hands habitually after touching bags, clothes, and surfaces (70.2%), after coughing and sneezing (70.6%), after shaking hands (66.1%), and before and after touching their eyes, nose, mouth, or face (66%). More than half the participants (55.2%) reported relying on alcohol as an alternative to water and soap for cleaning their hands on occasion. Most participants (76.5%) reported drying their hands using a tissue or a clean towel after washing them. One-third of the population (36.5%) reported preparing food occasionally even when sick (with symptoms of flu, cold, diarrhea, and cough, among others). Almost half the population (49%) reported that they never shared plates, spoons, or other utensils with family members while eating, whereas a small percentage of the population (12.5%) reported engaging in this practice (Table 3 - see PDF). The mean score in this section was 28.84±3.16, with a maximum of 36 points (80%) obtained, which indicated overall good personal hygiene during the COVID-19 pandemic.

### Food preparation practices:

Approximately half of the participants (46.3%) reported washing fresh fruits and vegetables with water and vinegar/special vegetable washing solutions during the COVID-19 pandemic. Almost half of the participants reported thawing frozen food of animal origin on the kitchen counter, whereas only 14.5% of the population reported using the refrigerator for this purpose. Most participants (73.6%) reported using a different cutting board for cutting fruits. More than half of the participants (55.1%) reported cleaning the kitchen countertop, utensils, and equipment appropriately using a rag, detergent, and warm water (Table 4 - see PDF). The mean score in this section was 8.08±1.91, with a maximum of 12 points (67%) obtained, indicating moderate food safety practices during preparation.

### Association between socio demographic characteristics and food safety practices:

The association between the socio demographic characteristics and food safety practices are shown in Table 5(see PDF). Age was significantly associated with food purchasing habits during the COVID-19 pandemic (p<0.001), behaviour during grocery shopping (p<0.001), grocery unpacking (p=0.017), food preparation (p=0.002), and personal hygiene (p<0.001). Among the different age groups, older women, particularly women aged more than 55 years, received the highest scores in food purchasing habits, behaviour during grocery shopping, grocery unpacking, and food preparation (p=0.002). Women aged 36-45 years received the highest scores in questions on personal hygiene practices.

Marital status and presence of children were significantly associated with all dependent variables (p<0.001), except for behaviour during food unpacking (p=0.340 and p=0.059, for marital status and presence of children, respectively). Compared to single women and women without children, married women or women previously married (widowed and divorced), as well as women with children, showed better food purchasing habits and received better scores in behaviour during grocery shopping, food preparation, and personal hygiene. The domain of employment significantly affected the dependent variables analyzed (p<0.001 for all variables except behaviour during food unpacking, for which p=0.012). Retired women received the highest scores in changes in food purchasing habits during the COVID-19 pandemic, behaviour during grocery shopping, food unpacking, and food preparation at home. Unemployed women obtained scores similar to those obtained by retired women in the aforementioned domains. Participants working in the health domain received the highest scores in questions on personal hygiene.

The analysis revealed that monthly income was a significant factor affecting the scores for food purchasing habits (p=0.004), behaviour during grocery shopping (p<0.001), and personal hygiene (p<0.001). Participants with an average income (10000-15000 SR/month) received the highest scores in changes in food habits during the COVID-19 pandemic, followed by women with no/low incomes. Although women with lower (<5000 SR/month) incomes received the highest scores in behaviour during grocery shopping and personal hygiene, women belonging to the high-income group also received comparable scores in these categories. The diagnosis of chronic diseases significantly affected the scores received in all food safety domains, barring personal hygiene (p=0.682). Compared to their counterparts, women with chronic diseases received higher scores in food purchasing habits (p=0.024), behaviour during grocery shopping (p=0.049), grocery unpacking (p=0.001), and food preparation (p=0.049). However, no significant associations were observed between the presence of patients with chronic diseases at home and the studied domains. Lastly, the presence of elderly individuals at home significantly affected the behaviour during grocery shopping (p=0.044), and participants with elderly individuals at home scored better than their counterparts.

## Discussion:

### Knowledge on corona virus transmission and prevention:

The majority of Saudi women (62.5%) were aware that SARS-CoV-2 cannot be transmitted through food, while almost all of them (97.6%) acknowledged that proper hand hygiene (washing with water and soap for 40 s) can reduce the risk of COVID-19. Although there is no scientific evidence that SARS-CoV-2 can spread directly through food or via the human digestive tract, the virus has been confirmed to survive on surfaces, plausibly when heavily contaminated droplets fall on the surfaces; therefore, food items/food packages can be carriers of the virus. As individuals come in contact with food items habitually several times per day, local, national, and global authorities emphasize following appropriate food safety measures (such as proper hand hygiene) to minimize the spread of COVID-19 [3].

In our study population, the women mostly relied on the website of the Saudi MOH for suitable advice on the appropriate practices during the COVID-19 pandemic. Additionally, a significant percentage of women (45.5%) sought this information on social media, whereas only a small proportion of women (17%) relied on the advice of health practitioners. The role of social media in spreading awareness among this population was considerable, with Twitter (a social networking application frequently used by Saudis) being flooded with awareness campaigns launched by ministers, celebrities, and academics [15].

### Food purchasing habits:

We observed a change in the food purchasing habits in response to the COVID-19 outbreak in the surveyed population. The majority of participants (more than 90%) reported that they stopped ordering food items and beverages from restaurants, prepared more meals at home, and reported purchasing grocery online more frequently than before(30% of the women). Our data are in line with those from the Netherlands, where half of the surveyed population reported visiting grocery shops less frequently than usual; however, only 6.8% of the participants reported an increase in the frequency of availing online delivery during the pandemic [16]. Meanwhile in China, the consumers reported reducing their visits to the supermarkets (from 54% to 35%) and increasing the frequency of online shopping (from 11% to 38%) [17>]. One of the most important drivers of the behavioural changes observed was the increased fear of potential corona virus transmission among individuals, both in Saudi Arabia as well as in other countries. Fear with warnings for maintaining social and physical distancing and avoiding congested areas (including shops and retail stores) by the national and local authorities affected the lifestyle and habits of the individuals. Accordingly, measures for minimizing the contact between individuals (such as online food delivery systems or contact-free delivery services) were introduced as alternatives to direct food purchase [10], and their increased use was evident in our cohort as well. Women mostly reported purchasing the same quantities of canned, frozen, and pre-packed foods as before, whereas more than half of the women reported an increase in their purchase of fresh vegetables and fruits, indicating that Saudi women tended to change their dietary habits toward healthier choices; this was also reported in a study performed in Qatar, our neighbouring country [18]. The underlying motive might be to improve immune responses to potential corona virus infection, as also reported recently in an Italian study [19].

### Behaviour during grocery shopping:

The majority of women reported following recommendations for the appropriate behaviour during grocery shopping in the pandemic. They mostly reported wearing masks, maintaining a 2-meter distance, frequently sanitizing hands while shopping, and wiping the shopping basket or trolley with a sanitizer during shopping. Our results are in line with those from a survey conducted in the USA, according to which almost all participants (90%) reported wearing a mask the last time they had visited an outdoor or public space [20], as well as with results from a survey conducted in Jordan, in which more than half of the food industry workers reported complying with the 2-meter rule of physical distancing within the working area [21]. The use of face masks combined with social distancing is recognized as an effective non-pharmaceutical intervention that can flatten the epidemic curve and help combat COVID-19 [22]. The use of gloves, masks, disinfectants, and sanitizers is also recommended for preventing and minimizing the spread of COVID-19. These measures are particularly important while visiting some of the identified clusters of higher SARS-CoV-2 transmission, which include congested areas such as shopping malls, buses, and hospitals [23]. In addition, food items and food packaging materials can be carriers of the virus; therefore, it is of utmost importance to adhere to the guidelines proposed by public health authorities regarding frequent sanitization and disinfection of hands, especially during visits to food retail shops [10].

### Behavior during food unpacking:

A greater proportion of women reported following the recommended steps during grocery unpacking at home. These women reported unpacking bags separately and away from kitchen items, cleaning and sanitizing the surfaces used for unpacking, cleaning cans, and immediately storing frozen or perishable food items. However, a considerably low percentage of individuals (13.2%) reported storing raw meat correctly in the bottom shelf of the refrigerator. Similarly, only a low percentage of people are aware of appropriate measures for raw meat storage, as reported in surveys conducted in Greece (23.1%) [24] and northern Jordan (20%) [25]. In this domain of food safety practices, cross-contamination is a critical determinant, and to avoid this, raw meat should be stored only on the bottom shelf. Conversely, food workstations and items coming in contact with food (surfaces, utensils, materials, counters, conveyor belts, and packaging materials) are also surfaces via which food handlers potentially come in contact with the virus, and these surfaces may act as carriers of SARS-CoV-2. Therefore, proper cleaning and sanitation as well as separation of purchased goods are a vital step in reducing the risk of cross-contamination. This is also applicable to the lids of canned food containers, which should be wiped before usage [10].

### Food preparation:

Most of the participants (85%) in our study reported washing fresh fruits and vegetables appropriately either under running cold water or using water and vinegar/special vegetable cleaning solutions. These results are in line with those reported by other Saudi and Irish studies, in which approximately 92% and two-thirds of the participants, respectively, reported washing fruits and vegetables under cold running water [26,27]. Additionally, the majority of the enrolled women (86.4%) reported using a separate cutting board for raw meat and fresh fruits or properly cleaning it if they used the same board. Similar results were obtained in another survey on the Saudi population where about 90% of the participants reported the same [28]. However, a relatively high percentage (46.5%) of women reported thawing frozen food of animal origin on the kitchen counters. Our results are in line with those obtained by our group where 47% of surveyed Saudi women reported thawing frozen meat and poultry over the kitchen counter. In Italy, about 63% of consumers defrosted meat and fish at room temperature [29]. Next, 55% of the participants in our cohort reported cleaning kitchen countertops, utensils, and equipment as recommended (using a rag, detergent, and warm water), and this rate was higher than that reported in surveys conducted in north Jordan (approximately 30%) [25], Greece (32%) [24] and Canada (29.6%) [30]. Food safety measures to be followed during food preparation are recommended to avoid cross-contamination during storage itself, and the habitual cleaning and disinfection of kitchen equipment, countertops, utensils, and refrigerators are some of the necessary procedures that could facilitate the same [31]. Conversely, the thawing of frozen non-vegetarian food products in the refrigerator prevents the rapid growth of contaminating microorganisms as well as the acceleration in spoilage caused by these microorganisms [32]. Although it was reported that corona virus cannot survive cooking, if fresh food items are frozen without prior washing and scrubbing, the virus can survive for up to 2 years while remaining in storage. Therefore, food items should be washed and scrubbed prior to their usage or storage to prevent cross-contamination [10].

### Personal hygiene:

In our study, the majority of women claimed washing their hands primarily after visiting public places, before eating and handling food, and after unpacking grocery bags. Our findings are concordant with those of studies conducted in Ireland, in which 85% of the respondents reported washing hands after handling raw meat [26], and in Saudi Arabia, in which more than 80% of the respondents stated washing their hands properly before food preparation [27,28]. Meanwhile, in a study conducted in northern Jordan, approximately 50% of the respondents reported washing hands after touching their clothes and face [25]. Additionally, 49% of the participants in our cohort did not report sharing plates and utensils with other family members during mealtime; however, only 23.7% of them avoided preparing food while sick, which could potentially increase the risk of cross-contamination. Farahat et al. showed that while Saudi women were mostly aware that individuals with diarrhea, fever, sore throat, or flu should not prepare food for others, some of them reported engaging in this activity even when unwell [33]. Individuals have been urged to maintain proper personal hygiene, particularly prior to food handling, as the first line of defense against SARS-CoV-2 infection [34]. Proper hand washing with warm running water and soap is an essential step, and hand sanitizers should only be used as an additional measure, and not as a substitute [35]. In line with proper personal hygiene practices, individuals with certain symptoms of illness (flu, sore throat, or diarrhea) should avoid food preparation, since the infected individual acts as a carrier of the virus. Therefore, if the individuals are involved in food preparation for other family members, the unintentional mishandling of food or the contact between a contaminated respiratory droplet and food items or kitchen utensils can increase the risk of SARS-CoV-2 transmission [36] and the consequent spread of the disease among family members. The same applies to the sharing of utensils and plates during mealtime among family members, which may further increase the risk of cross-contamination. Since there are several asymptomatic individuals who are carriers of SARS-CoV-2 [37], it is particularly important that family members refrain from meal-sharing practices.

### Associations among socio demographic characteristics, personal characteristics, and food safety practices:

According to our findings, age and the presence of chronic diseases were significant factors influencing the behavior of Saudi women during the COVID-19 pandemic. Notably, in our cohort, women belonging to high-risk groups of severe COVID-19 and associated illnesses (elderly and retired women or women diagnosed with chronic diseases) made better food choices in terms of dietary preferences (preference for healthier options) and purchase of food items (preference for items that minimize the spread of SARS-CoV-2). Furthermore, these women also reported better practices during grocery shopping, food unpacking, and food preparation compared to those reported by younger individuals. Our results are in line with those reported in the studies by Farahat et al. and Arfaoui et al. in which age was a significant factor influencing the mean scores of women's food safety knowledge and practices, with older women receiving the highest scores [28,33]. Additionally, food safety knowledge has been reported to increase with age, implying that the younger population would benefit from additional education in this domain [25]. However, age is not an independent factor; rather, it is coupled with the presence of chronic diseases (cardiovascular disease or diabetes, among others) [38]. Therefore, these groups of individuals are specifically urged to comply with the recommended guidelines for minimizing the spread of the infection and preventing serious consequences and were the case in our cohort. Besides retired women, participants from other employment groups, such as the health domain, also showed greater awareness of food safety practices. This group paid the greatest attention to appropriate personal hygiene practices. These workers were particularly urged to follow the recommended guidelines related to personal hygiene to reduce the rate of pathogen transmission in hospitals in the first place, and to limit the spread of SARS-CoV-2 to other patients, staff, and family members [39].

Evidently, unemployed and retired women obtained similar scores in questions related to food purchasing, unpacking, and preparation. Although our observations were contrary to those of Farahat et al. who reported that working women obtained higher scores in food safety knowledge and practices [33], our results were concordant with those reported by Ayaz et al. who reported that housewives averaged with the highest mean score, even though this association was not significant [14]. We also observed that women with average and lower monthly incomes tended to make better food purchasing choices during the pandemic, while women with the lowest income exhibited better grocery shopping and personal hygiene practices. Our results are not concordant with other data published on Saudi populations, as no significant relationship between monthly income and food safety knowledge and practices was reported [14]. However, other studies have shown that individuals from higher income households expressed less concern about food safety, and therefore, were more likely to engage in high-risk practices [40,41]. Although one might expect that a higher income would correspond to increased awareness of food safety practices, we observed the opposite trend. It is plausible that compared to women with lower income, women with higher income tended to prepare fewer meals at home [42] (prior to the COVID-19 outbreak), which resulted in a lower mean score of food safety practices among this population. Additionally, participants with lower income may have a greater fear of potential SARS-CoV-2 infection and related consequences, and their financial limitations may positively affect their attitude toward the appropriate food safety practices.

Lastly, adherence to food safety practices among women depended significantly on whether they are currently living or have lived with other family members (partners or children). In our cohort, married, divorced, widowed, and women with children received higher scores in food safety practices than their counterparts, which is in line with a previous study published by our group [28]. One proposed reason for our finding might be that single women have lower awareness of food safety as they are less likely to be engaged in cooking for themselves [43]. It is encouraging that compared to single women (or women without children), the aforementioned groups of women in our cohort received better scores in food safety practices, as it implies that these women tend to follow the recommended guidelines related to hygiene and food handling/purchase, which minimizes the chances of the potential spread of SARS-CoV-2 to their family members.

### Limitations of the study:

The study only targeted women as the primary domestic food handlers in Saudi Arabia; therefore, these findings cannot be applied to the general population or to other categories of domestic food handlers excluded from the study. Further, the data obtained in this study are based on self-reported habits/behaviors and practices of the participants; therefore, the observed results might be subject to overestimation of actual practices. The observed associations between certain socio demographic characteristics and food safety practices might have been overestimated as the sample sizes were limited in certain categories (such as widowed women, women with age more than 55 years, and women with high monthly incomes). Lastly, owing to ongoing pandemic, the questionnaire was distributed online, and therefore, it mostly excluded individuals with limited access to social media or with lower levels of education.

## Conclusion:

The obvious change in the food purchasing habits of the participants was characterized by a shift from outdoor to indoor meal preparation owing to the COVID-19 outbreak. We observed that the majority of women complied with the recommendations during grocery shopping, while more than half of the women followed the same rules while unpacking groceries at home. Approximately half of the participants reported following the recommended food preparation and personal hygiene practices; however, this was not applicable in all cases. During the pandemic, age, marital status, presence of children, professional engagement, monthly income, and presence of chronic diseases significantly affected the personal hygiene practices and the behaviour during grocery shopping, grocery unpacking, and food preparation among women. Women belonging to high-risk groups of severe COVID-19-related illnesses received higher food safety scores, implying that women from low-risk groups could benefit from additional education in this field. The variability observed among women with different socio demographic characteristics in terms of food safety behaviour and practices indicated that customized public education programs will be useful for the overall improvement of food safety practices and prevention of COVID-19 transmission resulting from the mishandling of food items.

## List of abbreviations:

COVID-19, corona virus disease; SARS-CoV-2, severe acute respiratory syndrome corona virus 2; WHO, World Health Organization.

## Authors' contributions:

Leila Arfaoui was responsible for conceptualization, methodology, data curation, writing-reviewing & editing. Wejdan Alghafari was responsible for conceptualization, methodology, data curation, writing original draft. All authors have read and approved the final manuscript.

## References

[1] Chu DK (2020). Lancet.

[2] Wolfel R (2020). Nature.

[3] Duda-Chodak A (2020). Trends Food Sci Technol..

[5] Van Doremalen N (2020). N Engl J Med..

[6] Klein G (2004). Dtsch Tierarztl Wochenschr..

[7] Bergeron JG (2016). IBM Journal of Research and Development.

[8] Ceylan Z (2020). Virus Disease.

[9] Yepiz-Gomez MS (2013). Food Environ Virol..

[10] Olaimat AN (2020). Front Microbiol..

[11] https://www.openepi.com/SampleSize/SSPropor.htm.

[12] https://foodinsight.org/consumer-survey-covid-19s-impact-on-food-purchasing/.

[13] Desai AN, Aronoff DM (2020). JAMA.

[14] Ayaz WO (2018). Foods.

[15] Algaissi AA (2020). J Infect Public Health.

[16] Poelman MP (2021). Appetite.

[17] Li J (2020). Tijdschr Econ Soc Geogr..

[18] Ben Hassen T (2020). Sustainability.

[19] Scarmozzino F, Visioli F (2020). Foods.

[20] Knotek E (2020). Economic Commentary.

[21] Omar SS (2020). Eurasian Journal of Biosciences.

[22] Li T (2020). PLoS One.

[23] Ong S (2020). JAMA.

[24] Lazou T (2012). Food Control.

[25] Osaili TM (2011). Food Control.

[26] Moreb NA (2017). Food Control.

[27] Sharif L, Al-Malki T (2010). Food Control.

[28] Arfaoui L (2021). Current Nutrition & Food Science.

[29] Langiano E (2012). Journal of public Health.

[30] Burke T (2016). Food Control.

[31] NG Marriott (2018). Principles of Food Sanitation.

[32] Badrie N (2006). Food Control.

[33] Farahat MF (2015). Food Control.

[34] Alzyood M (2020). J Clin Nurs..

[35] Rundle CW (2020). J Am Acad Dermatol..

[36] https://www.who.int/.

[37] Li Y (2020). Front Microbiol..

[38] Romero Starke K (2020). Int J Environ Res Public Health.

[39] Lotfinejad N (2020). J Hosp Infect..

[40] Nesbitt A (2009). J Food Prot..

[41] Nesbitt A (2014). Food Control.

[42] Roseman M, Kurzynske J (2006). J Food Prot..

[43] Nguyen ATL (2018). Int J Environ Res Public Health.

